# An Incentive Mechanism in Mobile Crowdsourcing Based on Multi-Attribute Reverse Auctions

**DOI:** 10.3390/s18103453

**Published:** 2018-10-14

**Authors:** Ying Hu, Yingjie Wang, Yingshu Li, Xiangrong Tong

**Affiliations:** 1 School of Computer and Control Engineering, Yantai University, Yantai 264005, China; gaoyang@ytu.edu.cn (Y.H.); txr@ytu.edu.cn (X.T.); 2 School of Department of Computer Science, Georgia State University, Atlanta, GA 20202, USA

**Keywords:** multi-attribute reverse auction, malicious competition, dynamic threshold, crowdsourcing, online incentive mechanism

## Abstract

In order to avoid malicious competition and select high quality crowd workers to improve the utility of crowdsourcing system, this paper proposes an incentive mechanism based on the combination of reverse auction and multi-attribute auction in mobile crowdsourcing. The proposed online incentive mechanism includes two algorithms. One is the crowd worker selection algorithm based on multi-attribute reverse auction that adopts dynamic threshold to make an online decision for whether accept a crowd worker according to its attributes. Another is the payment determination algorithm which determines payment for a crowd worker based on its reputation and quality of sensing data, that is, a crowd worker can get payment equal to the bidding price before performing task only if his reputation reaches good reputation threshold, otherwise he will get payment based on his data sensing quality. We prove that our proposed online incentive mechanism has the properties of computational efficiency, individual rationality, budget-balance, truthfulness and honesty. Through simulations, the efficiency of our proposed online incentive mechanism is verified which can improve the efficiency, adaptability and trust degree of the mobile crowdsourcing system.

## 1. Introduction

In recent years, the development of smart devices (e.g., smart mobile phones, smart watches, etc.), has led to a new paradigm for data collection and problem solving. According to the International Data Corporation (IDC), the total number of smart mobile phone users in the world will amount to 2.53 billion at the end of 2018, which accounts for about 36% of the global population. This indicates that there are a large number of potential participants for crowd-sensing applications.

At present, most smart devices are equipped with a richness of embedded sensors (e.g., accelerometers, direction sensors, gyro-sensors, temperature sensors, GPS (Global Positioning System), cameras, etc.) [1,2]. Along with smart devices’ users round-the-clock, these smart devices with powerful sensing capabilities can interact with the surrounding environment, so users with smart devices may collect sensing data for sensing tasks. Certainly, the users with smart devices have the right to select the appropriate sensing task based on their locations, preferences and sensing capabilities. According to the aforementioned, mobile crowdsourcing applications can achieve varies functions such as environmental monitoring, traffic monitoring, health care and convenience services.

However, the mobile crowdsourcing systems (MCSs) can perform properly and obtain benefit only if a large number of users participate in the sensing task by using their smart devices [3]. However, there are three main reasons for the current low participation rate. First, there is a lack of appropriate incentive mechanisms to motivate users’ participation. Second, performing sensing tasks may consume some resources, such as equipment battery power and network resources. Finally, the sensing data are submitted by users, which will reveal their privacy [4,5,6], i.e., trajectory information [7], daily routines, etc. This will hinder the development of mobile crowdsourcing applications. Therefore, it is urgent to propose effective incentive mechanisms to motivate the participation of users.

Most of the platform-centric incentive mechanisms adopt the auction models [8] and micro-payment methods. In crowdsourcing system, requesters are buyer and crowd workers are seller. When a requester requests a task, crowd workers can bid this task by submitting the bidding profile. The advantage of auction model is that can discover prices for buyer and seller, which can effectively control the incentive costs. However, the existing auction-based model has the following problems.

(1) Most auction models are designed to win the auction at the lowest price. However, many researchers fail to consider the unfairness caused by malicious competition, i.e., they upload bids lower than their cost for winning the auction and improve their utility.
(2) Most payments for sensing tasks are ex-ante [9,10], which means that the crowd workers are paid before they perform the sensing tasks. Due to the selfishness of individuals, some crowd workers may not perform the task truthfully after receiving payment, which is known as free-riding [11] and that will result in low quality sensing data.
(3) The general auction models only consider the interests and preferences of crowd workers, however, they ignore the task requesters’ requirements of crowd workers.
(4) Most researchers only consider the price attribute in the auction process, which can better ensure the budget balance. However, by only considering the price, they ignore the impact of other attributes, which cannot ensure the quality of sensing data.

In order to solve the above problems, this paper designs an incentive mechanism based on auction through combining a reverse auction and a multi-attribute auction. The main contributions of this paper are summarized as follows:

(1) An incentive mechanism based on the combination of reverse auction and multi-attribute auction is designed to address the crowd workers’ malicious competition behavior in price bidding. Through updating the reputation and trust degree of crowd workers based on their performance, the free-riding problem is addressed [12,13].
(2) Adopt the dynamic threshold of multi-attribute reverse auction algorithm to select the qualified crowd workers. Different from other payment schemes [14], our proposed payment scheme considers both the reputation of crowd workers and the quality of sensing data, which can inspire crowd workers to submit high-quality sensing data and improve their reputations.
(3) Experimental results prove that our proposed incentive mechanism can achieve computational efficiency, individual rationality, budget-balance, truthfulness, and honesty.

The rest of this paper is organized as follows: in Section 2, we overview the incentive mechanism based on an auction process in MCSs. In Section 3, we describe the system model and the proposed incentive mechanism. Section 4 evaluates the performance and analyses the result of the mechanism through simulations and experiments. Finally, we conclude the paper in Section 5.

## 2. Related Works

The auction-based incentive mechanism is the main method in reward incentive. At present, the main auction algorithms include reverse auction (RA), multi-attribute auction (MAA), all-pay auction (AA), two-stage auction (TA), combinatorial auction (CA), double auction (DA), vickrey-Clarke-Groves (VCG) auction and their various combinations.

RA is an auction with multiple sellers and one buyer. The advantage of RA is that it can avoid the exiting of users and cost explosions. Lee et al. [15] first proposed a RA-based incentive mechanism for mobile crowdsourcing. Compared with the previous fixed-price payment, the RA-based incentive mechanism dynamically selects participants based on their trust degrees to avoid participants losing confidence and dropping out from MCSs. Meanwhile, it may guarantee the participation rate while minimizing payments. However, this mechanism did not consider the truthfulness of crowd workers and the quality of sensing data, which will generate free-riding problems in MCSs. Peng et al. [16] designed a data quality-based incentive mechanism that considered the quality of sensing data, rewards and contributions to inspire crowd workers to provide high quality sensing data. Yang et al. [17] considered two system models: the crowdsourcer-centric model using a Stackelberg game and the user-centric model using a reverse auction to design incentive mechanisms. Zhao et al. [18,19] adopted a reverse auction to provide real-time online incentives. According to the problems of malicious competition and free-riding problems in MCSs, Zhu et al. [20] combined RA and Vickrey auction to propose the reverse-Vickrey auction (RVA). The mechanism is designed so that the bidder with the second-lowest bidding price will win the auction, which can avoid malicious competition, i.e., a bidding price lower than the actual cost, to guarantee the fairness of auctions. However, the bidder with the lowest bidding price is not necessarily a malicious competitor, which may generate a new unfairness problem in RVA. 

MAA is an auction where buyers and sellers make multiple negotiations on bidding prices and other attributes. Krontiris et al. [21] adopted a MAA-based incentive mechanism to inspire crowd workers to participate in sensing tasks and provide high quality sensing data. In this incentive mechanism, participants will improve the quality of their sensing data based on the feedback of auction results for increasing their bidding prices in future. Then Albers et al. [22] proposed coupons through combining with multi-attributive auction to inspire user participation, which can also inspire more people in the target sensing area and increase the overall utility of data for service providers. 

AA means that the platform only pays the crowd worker with the largest contribution, not all participants. Luo et al. [23,24] proposed an AA-based approach to inspire agents to act for maximizing principal’s profit while allowing agents to reap strictly positive utility, and then adopted AA to solve the problem of heterogeneous crowdsourcing. 

TA means that the first batch of crowd workers as a sample participating in the auction which be used to make an informed decision on whether to accept the remaining crowd workers. The platform automatically rejects the first batch of crowd workers, which is unfair to the crowd worker who arrives early. Wang et al. [25] proposed an improved TA-based incentive mechanism to select crowd worker candidates statically, and then dynamically select winners after bidding, which overcomes the unfairness problem and motivate users to arrive in time. Then Wang et al. [26] proposed the improved TA auction algorithm based on trust degree and privacy sensibility (TATP) with location privacy-preserving and the k-epsilon-differential privacy-preserving to prevent users’ location information from being leaked.

Xu et al. [27] adopted reverse combinatorial auction and added the quality of information into the incentive mechanism to achieve approximate maximum of social welfare. Jin et al. [28] adopted DA-based incentive mechanism allows multiple requesters to compete for crowd workers’ resources to encourage the participation of data requesters and crowd workers. Chen et al. [29] proposed a novel truthful double auction mechanism named TDMC for a two-sided heterogeneous MCS market. Yang et al. [30] proposed a k-anonymity auction as a single-round sealed-bid double auction to design incentive mechanism for k-anonymity location privacy.

Gao et al. [31] proposed a Lyapunov-based VCG auction and designed the incentive mechanism from two aspects (time-dependent and location-aware) for encouraging the long-term participation of participants. Duan et al. [32] proposed two truthful auction mechanisms for different working patterns to minimize social cost, which a VCG-based auction mechanism is suited for the continuous working pattern, and the suboptimal auction mechanism is suited for the discontinuous working pattern.

Han et al. [33] proposed a Lyapunov optimization based on a decision support approach, the reputation-aware task subdelegation approach with dynamic worker effort pricing (RTS-P) to address the fact that spontaneous evolution of the complex resource allocation dynamics may lead to undesirable herding behaviors. They proposed a surprise-minimization-value maximization (SMVM) approach to address the NP-hard problem of allocation task to worker and maximize social welfare in crowdsourcing system [34]. Moreover, they proposed the concept of a worker desirability index (WDI) and increase the collective productivity by evaluating WDI to influence individual workers in real time about courses of action.

However, most of the aforementioned works ignored the requirements of the task requesters for crowd workers (e.g., current location [35], the distance to target area, trust degree, privacy sensitivity, sensing time, reputation, etc.). These requirements will have a greater impact on data quality. Therefore, multi-objective optimization algorithms were researched by scholars [36]. In this paper, we combine multi-attribute reverse auction and dynamic threshold to select crowd workers for different types of tasks. Furthermore, we determine the payments based on the data quality, and the crowd workers with high reputation will get payment before performing tasks. Therefore, it can inspire crowd workers to provide high quality sensing data in order to improve their reputations [37].

## 3. The Proposed Incentive Mechanism

**Figure 1:**
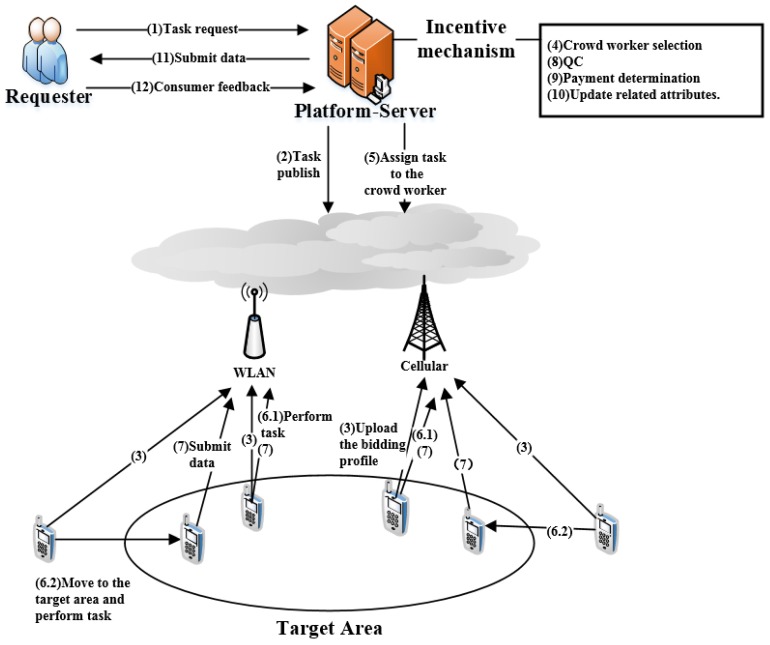
The framework of a mobile crowdsourcing system.

The process of MCS in our model is shown by Figure 1. The system comprises three roles, which include task requesters, crowd workers and service platform. The platform includes many sensing servers in a cloud, and crowd workers can interact with the platform through wireless local area networks (WLANs) or cellular networks. The mobile user who sends a task request to the platform is a task requester, and the mobile users who perform the task are crowd workers. 

Firstly, a requester requests a sensing task, which includes some task requirements (e.g., deadline, budget and worker’s context), and sends this request to the platform to recruit suitable crowd workers to complete this task (Step 1). The platform issues the task to crowd workers within the location scope of the task that can be completed before the deadline (Step 2). Crowd workers within the location scope select interested tasks and submit bidding profiles (e.g., interested tasks, bidding price, location, distance, reputation, trust degree, etc.) to the platform (Step 3). The platform selects suitable crowd workers based on the bidding profiles submitted by crowd workers and assigns tasks (Steps 4, 5). The crowd workers perform the sensing tasks and then upload the sensing data to the platform (Steps 6, 7). The platform determines the payment for crowd workers after quality certifying for the sensing data, then updates their related attributes based on their performances (Steps 8, 9, 10). The platform sends the sensing data to requester. After receiving the sensing data, the requester pays for the crowd worker based on the data quality (Steps 11, 12). In this paper, we mainly research the following two aspects: (1) crowd worker selection; (2) payment determination.

### 3.1. System Model

In this model, crowd workers can select multiple tasks to perform, and tasks can also be performed by multiple crowd workers. This paper combines multi-attribute auction and reverse auction to design the online auction method. The platform has some heterogeneous sensing tasks in specific areas submitted by task requesters. The corresponding descriptions for frequently used notations are shown in Table 1. The set of sensing tasks in one time slot is presented by $\Gamma =\{\tau_{1},\tau_{2},\ldots ,\tau_{j},\ldots ,\tau_{n}\}$, where $\tau_{j}$ indicates the *j*-th task. The budget of $\tau_{j}$ is represented by $B_{j}$. $W=\{w_{1},w_{2},\ldots ,w_{i},\ldots ,w_{m}\}$ represents the set of crowd workers, where $w_{i}$ denotes the *i*-th crowd worker. According to the specific area, task requester claims the requirements for crowd workers (e.g., sensing time, location, reputation, trust degree, etc.). Then the platform sets the attribute’s thresholds for $\tau_{j}$, which denoted by $\theta_{j}=\left\{\theta_{l},\theta_{d},\theta_{t},\theta_{tr},\theta_{b},\theta_{rp},\theta_{pt}\right\}$ based on historical information, where $\theta_{l}$ denotes the threshold of location which is a range of target area, $\theta_{d}$ represents the threshold of distance, $\theta_{t}$ indicates the threshold of sensing time, $\theta_{tr}$ means the threshold of trust degree, the threshold of bidding price denoted by $\theta_{b}$ and the threshold of reputation and the possibility to target area represented by $\theta_{rp}$ and $\theta_{pt}$ respectively. The thresholds will be updated dynamically. After completing $\tau_{j}$, $w_{i}$ will bring a fixed profit $v_{j}$ to the requester. According to the personal preferences and their own conditions, the arriving crowd workers select the interested task, and then online submit their bidding profiles to the platform. $F_{i}=\{\Gamma_{i},Bid_{i},A_{i}\}$ is the bidding profile of $w_{i}$, where $\Gamma_{i}\subseteq \Gamma$ denotes the interested task set of $w_{i}$. $Bid_{i}=\{b_{i1},b_{i2},\ldots ,b_{ij},\ldots ,b_{im}\}$ denotes the bidding price set, which includes the bidding price $b_{ij}$ submitted by $w_{i}$ for $\tau_{j}$. $A_{i}=\left\{l_{i},d_{i},t_{i},tr_{i},rp_{i},pt_{i},pr_{i}\right\}$ denotes the attribute set of $w_{i}$, where $l_{i}$ is the location of $w_{i}$, $d_{i}$ denotes the distance of $w_{i}$ to the target area, $t_{i}$ is $w_{i}$’s sensing time and the crowd worker must finish the sensing task and upload the sensing data to the platform within the time range $t_{i}$. The parameter $tr_{i}$ indicates $w_{i}$’s trust degree, $rp_{i}$ means $w_{i}$’s reputation value, $pt_{i}$ denotes the possibility that $w_{i}$ will move from the current location to the target area, and $pt_{i}$ denotes $w_{i}$’s privacy sensitivity. The platform receives the bidding profile submitted by the crowd worker and compares the crowd worker’s attributes with the system threshold. When all the attributes of this crowd worker satisfy the threshold requirements, and the remaining budget of task auctioned by the crowd worker is sufficient, the crowd worker will be accepted and assigned the task. Finally, the platform removes $\tau_{j}$ from the task list when it is completed. The parameter $c_{ij}$ means the cost of $w_{i}$ for completing task $\tau_{j}$, which includes equipment electric, cost of transport and network consumed by the crowd worker for performing the task. Then a reward will be paid when the micro-task is completed by $w_{i}$, which is calculated by Equation (1):

(1) $$p_{ij}=\{\begin{array}{cc} b_{ij} & ,rp_{i}\geq \theta_{rp}^{good}\\ b_{ij}\cdot (\lambda \cdot e^{q_{ij}}-1) & ,otherwise \end{array}\;$$

where $\theta_{rp}^{good}$ denotes the fixed system threshold of good reputation, which given based on the historical information fed back by the requester and $\theta_{rp}^{good}>\theta_{rp}$. Furthermore, the crowd worker who has good reputation can get payment before performing tasks. The parameter $q_{ij}$ indicates the data quality of the sensed data submitted by $w_{i}$. λ is the system parameter, where $\frac{c_{ij}+b_{ij}}{b_{ij}}\cdot \frac{1}{e^{q_{ij}}}\leq \lambda \leq \frac{2}{e^{q_{ij}}}$ in order to the total payment $P_{j}$ to satisfy following constraint: $P_{j}\leq B_{j}$, where $P_{j}=\sum_{i=1}^{n_{j}}p_{ij}$.

Therefore, the utility of $w_{i}$ after completing $\tau_{j}$ is defined by Equation (2):

(2) $$u_{ij}=p_{ij}-c_{ij}\;$$

and the utility of the platform is defined by Equation (3):

(3) $$U=\sum_{\tau_{j}\in \Gamma_{i}(W_{j})}v_{j}-\sum_{\tau_{j}\in \Gamma_{i}(W_{j})}P_{j}\;$$

where $\Gamma_{i}(W_{j})$ denotes the set of sensing tasks that all crowd workers have finished.

According to the incentive mechanisms proposed by [9,20], the online incentive mechanism should satisfy the following five properties:

*Computational Efficiency*: an online incentive mechanism is computational efficiency if the whole process is completed in polynomial time.

*Individual Rationality*: an online incentive mechanism is individual rationality if the utility of each bidder is non-negative.

*Budget-Balance*: an online incentive mechanism is budget-balance if the utility of a requester is non-negative.

*Truthfulness*: an online incentive mechanism is truthful if no bidder can increase its utility by submitting a bidding price that deviates from the true value regardless of the bidding prices of others.

*Honesty*: the crowd worker must be a crowd worker who really wants to perform a sensing task, and there are not malicious competitions on bidding price.

### 3.2. Online Auction

In order to decide immediately to whether or not to accept the crowd worker, this paper adopts dynamic threshold to select crowd workers based on the bidding profiles submitted by crowd workers. That is to say, the crowd worker will be selected to sense the task if his attributes satisfy the threshold standard. The initial threshold is determined based on the historical information. Furthermore, for adapting different situations, the threshold changes dynamically, so that suitable high-quality crowd workers can be selected in different situations. The dynamical threshold is calculated by Equation (4):

(4) $$\theta_{i}=\{\begin{array}{cc} \frac{\theta_{i-1}+(1+\rho )\cdot attributes_{i}}{2} & ,attributes_{i}<\theta_{i-1}\\ \theta & ,attributes_{i}=\theta_{i-1}\\ \frac{\theta_{i-1}+(1-\rho )\cdot attributes_{i}}{2} & ,attributes_{i}>\theta_{i-1} \end{array}\;$$

where $\theta_{i}$ denotes the new threshold after *i*th crowd worker’s attributes join in and the initial threshold denoted by $\theta_{0}$ which is set based on history information. The $attributes_{i}$ indicates the value of *i*-th crowd worker’s attributes. The parameter ρ means the system parameter that adjusts the threshold change and ρ∈[0,1). There are three cases when the threshold is updated. The first is that the bidder’s attribute value is greater than the current threshold, the attribute value is appropriately adjusted smaller than before and the new threshold is the average of the adjusted attribute value and the current threshold. If the two are equal, the threshold does not change. Otherwise, the attribute value is appropriately adjusted larger than before and the new threshold is the average of the adjusted attribute value and the current threshold. Therefore, when the attribute value of the crowd worker is small, the system can dynamically reduce the threshold, otherwise, the threshold can be dynamically increased.

**Figure 2:**
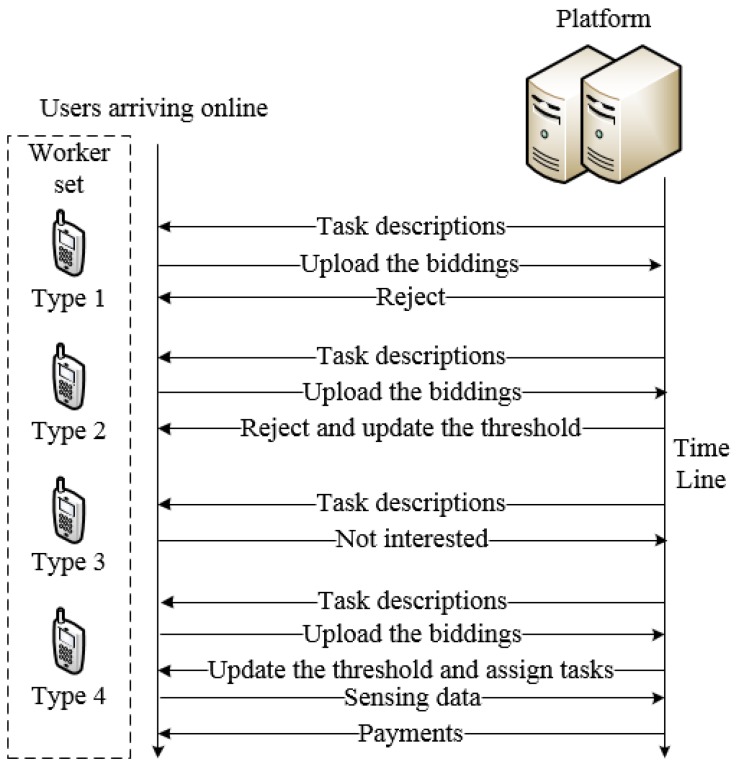
The processing procedure for the proposed incentive mechanism.

However, because of the inherent drawbacks of dynamic threshold, it is easy for a malicious user to change the threshold by submitting unreasonable price. For preventing this phenomenon, the platform will evaluate the reasonableness of the bidding price based on crowd worker’s attributes and historical information when receiving the bidding profile submitted by crowd worker. Then, the threshold is updated by the platform. There are four possible situations between platform and crowd workers in one transaction, which are shown in Figure 2. 

The specific processes are as follows:

(1) The platform issues tasks for crowd workers, then an interested crowd worker submits his bidding profile to the platform. After evaluating his bidding price and historical information the platform rejects this crowd worker because he is considered a malicious competitive bidder.
(2) The platform issues tasks for crowd workers, then an interested crowd worker submits his bidding profile to the platform. The platform rejects this crowd worker because his attribute values do not satisfy the requirements, then updates the attribute thresholds.
(3) The platform issues tasks for crowd workers, but the crowd worker is not interested in this task.
(4) The platform issues tasks for crowd workers, then an interested crowd worker submits his bidding profile to the platform. The platform accepts the crowd worker, and updates the attribute thresholds, then assigns the task for him. After completing the task, the crowd worker submits his sensed data to the platform.

**Figure 3:**
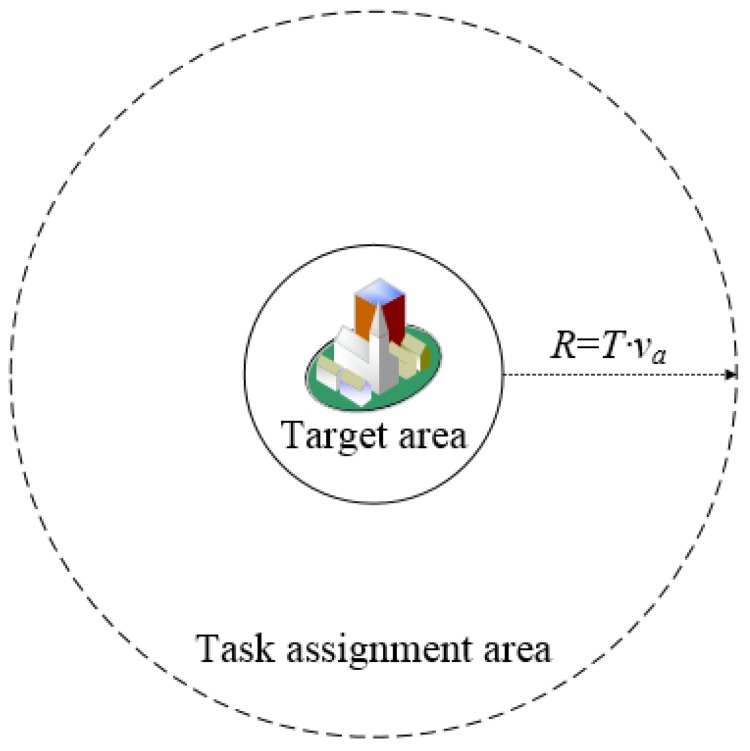
Task assignment area relation map.

The platform publishes the task to crowd workers within a certain range. The platform can select suitable crowd workers only if there are crowd workers bidding for the task, else it will not be executed by crowd workers. Due to the heterogeneous distribution of crowd workers [38,39,40], if the task is released only for the crowd workers in the target area, there may be a problem that the crowd workers are insufficient. In order to avoid this problem, the task publishing area is shown in Figure 3. The area is centered on the target area, according to the average moving speed of the crowd worker and the planned time of the task, the platform publishes tasks for the crowd workers in the area based on their attributes. The task publishing radius is shown by Equation (5):

(5) $$R=T\cdot v_{a}\;$$

where T is the duration of a task, that is, the deadline of the task minus the current time. The parameter $v_{a}$ is the normal moving speed of a crowd worker, and R denotes the radius centered on the target area.

For obtaining high-quality sensing data to enable crowdsourcing system to provide better services, this paper considers the following attributes when selecting crowd workers:

(1) *Reputation*: the crowdsourcing system will give a base value of reputation for every new crowd worker, and then we use Gompertz function [41] to update the reputation scores. Gompertz function is a type of growth curve function model which describes the three stages of the occurrence, development and maturity of things, and the development speed of each stage is different. We select this function to update the value of reputation and trust degree, because it is more suitable to model the concept of reputation and trust degree in human interactions. The Gompertz function defined by Equation (6).
  (6) $$f(x)=\omega \cdot e^{\alpha \cdot e^{\gamma \cdot x}}\;$$
  where ω, α and γ are function parameters. Specifically, ω specifies the upper asymptote of this function, α controls the displacement along the x axis and γ adjusts the growth rate of the function [41]. In Equation (6), x is the variable the Gompertz function. We get $rp_{i}$ through replacing x with $x_{i}$, which is explained and defined below. $rp_{i}$ is calculated by Equation (7):
  (7) $$rp_{i}(x_{i})=\omega \cdot e^{\alpha \cdot e^{\gamma \cdot x_{i}}}\;$$
  In this paper, we design $rp_{i}$ to reflect the average level of historical information, which be used as an indication for the possibility that the crowd worker is cost-effective in this time and future. The input of Equation (7) needs to reflect the historical information which includes task quality completed by the crowd worker as well as the task value and the bidding price. In particular, we hope the high value of reputation could represent the crowd worker usually bidding a task with high value in a lower price and complete the task in high quality. However, affected by the time factor, the task’s information that is closer to the current task has a greater impact on the crowd worker’s reputation and we represent this time delay based on the Ebbinghaus forgetting curve [42] in psychology. The input of Equation (7) is determined by Equation (8):
  (8) $$x_{i}=\frac{rp_{0}+\sum_{j=1}^{n_{i}^{total}}q_{ij}{}^{\frac{b_{j}}{v_{j}}}\beta_{j}}{1+\sum_{j=1}^{n_{i}^{total}}\beta_{j}}\;$$
  where $rp_{0}$ is the base value of reputation depends on crowdsourcing system and is the same for every new crowd worker. The parameter $\beta_{j}$ is the time decay factor in the *j*th task sensing based on Ebbinghaus forgetting curve which is the law of human brain when forgetting new things. As time passes, the impact of historical task performed by $w_{i}$ gradually diminishes until it tends to 0, as shown by Equation (9):
  (9) $$\beta_{j}=\{\begin{array}{cc} 1 & ,j=n_{i}^{total}\\ e^{-\frac{1}{j}} & ,1\leq j<n_{i}^{total} \end{array}\;$$
(2) *Trust degree*: the crowdsourcing system will give a base value of trust degree for every new crowd worker and then we use the same idea as reputation to update the trust degree, which is shown by Equation (10)
  (10) $$tr_{i}(y_{i})=\omega \cdot e^{\alpha \cdot e^{\gamma \cdot y_{i}}}\;$$
  where $y_{i}$ is the input of Equation (10) and $tr_{i}$ grows as $y_{i}$ grows.
  However, different from $rp_{i}$, we hope $tr_{i}$ could reflect the overall situation in which the crowd worker completed tasks in the past. Therefore, $y_{i}$ needs to reflect the tasks’ quality of a crowd worker has completed. The more tasks with good-quality, the greater the $y_{i}$ and $tr_{i}$. In contrast, the trust degree of crowd worker is low if he has done many tasks with bad-quality in the past. The good-quality and bad-quality are distinguished by the system. The crowd worker should do the new task with the quality no less than before if he wants to improve his trust degree. $y_{i}$ can be calculated by Equation (11):
  (11) $$y_{i}=\frac{tr_{0}+n_{i}^{good}\sum_{j=1}^{n_{i}^{good}}q_{i}^{j}\beta_{j}+n_{i}^{bad}\sum_{j=1}^{n_{i}^{bad}}q_{i}^{j}\beta_{j}}{1+n_{i}^{total}\sum_{j=1}^{n_{i}^{total}}\beta_{j}}\;$$
  where $tr_{0}$ is the base value of trust degree depends on crowdsourcing system and is the same for every new crowd worker.
(3) *Location*: the current location of a crowd worker when he submits the bidding profile, $w_{i}$’s location is expressed as $l_{i}=(x_{i},y_{i})$.
(4) Distance: the shortest distance that a crowd worker moves from the current location to the target area, which is expressed by $d_{i}$.
(5) The possibility that a crowd worker moves to the target area: according to the crowd worker’s historical behavior information, the probability that the crowd worker moves from the current location to the target area is computed by Equation (12):
  (12) $$pt_{i}=\{\begin{array}{cc} \frac{m_{t}}{m_{c}} & ,m_{c}\neq 0\\ 0 & ,m_{c}=0 \end{array}\;$$
  where $m_{c}$ denotes the total times that $w_{i}$ came to the current area, and $m_{t}$ represents the times that $w_{i}$ moved from the current area to the target area.
(6) *Privacy sensitivity*: this attribute affects the crowd worker’s choice of tasks and the payment expectations [43]. When a crowd worker selects a task, he will judge the privacy requirement based on his privacy sensitivity level. The privacy sensitivity of $w_{i}$ is represented by $pr_{i}$.
(7) *Sensing time*: affected by the current location of a crowd worker and the device held by the crowd worker. The cost of a crowd worker increases with the increase of the sensing time. The sensing time is represented as $t_{i}=d_{i}-a_{i}$, where $a_{i}$ indicates the start time that $w_{i}$ plans to perform the task, $d_{i}$ denotes the time when the crowd worker submits sensed data.
(8) *Bidding price*: the reserve price that $w_{i}$ wants to sell his sensed data. The bidding price of $w_{i}$ for $\tau_{j}$ is expressed by $b_{ij}$.

Because of the budget constraint, a bidder who becomes the crowd worker of $\tau_{j}$ should not only meet the above attribute requirements, but also satisfy the condition shown by Equation (13):

(13) $$\sum_{i=1}^{n_{j}}b_{ij}\leq B_{j}\;$$

where $n_{j}$ denotes the number of crowd workers for $\tau_{j}$.

Algorithm 1 describes the process of selecting crowd workers. At one moment, a crowd worker arrives at and submits a bidding profile. The platform judges whether the crowd worker is in the target area based on its location attribute. If it is true (lines 3), the user’s cost will be calculated whether equals to the bidding price according to the cost estimation formula and the calculation error *ɛ* (lines 7). If so, it can be inferred that there is no malicious competition on bidding price, and then it is determined whether other attributes (distance, sensing time, reputation, trust degree, bidding price) satisfy the threshold requirements (lines 8). If it is true, we further check whether the remaining budget of this task is sufficient for paying this crowd worker (lines 9). And the task will be assigned to this crowd worker if the budget is sufficient (lines 10). Then, the system attributes’ threshold will be updated accordingly (lines 11). However, if the user is not in the target area (lines 20), we further check whether the probability of the user will move to the target area satisfies the system threshold (lines 24), which is a fixed value. Then we can continue the 4–18 steps (lines 25) if it is satisfied.

**Algorithm 1** Crowd Workers Selection**Input:**$w_{i}$’s bidding profile $F_{i}$, task set Γ, $\tau_{j}$’s budget $B_{j}$, the initial threshold set $\theta_{j}=\left(\theta_{lj},\theta_{b0},\theta_{t0},\theta_{tr0},\theta_{rp0},\theta_{d0},\theta_{pt}\right)$**Output:** the crowd worker set $W_{j}$ of $\tau_{j}$1: **for**
i←1
**to**
m
**do**2:  **for**
j←1
**to**
$n_{i}$
**do** //each task that is submitted by $w_{i}$3:    **if**
$w_{i}$ in the target area **then**4:    **if**
$b_{ij}+\varepsilon \neq {\mathrm{cost}}_{ij}$
**then** //malicious competition in bidding price5:      **continue**6:    **end if**7:      **if**
$b_{ij}+\varepsilon ={\mathrm{cost}}_{ij}$
**then**8:    **if**
$b_{ij}\leq \theta_{b},t_{ij}\leq \theta_{t},tr_{i}\geq \theta_{tr},rp_{i}\geq \theta_{rp},d_{i}\leq \theta_{d}$
**then** //its attributes satisfy threshold requirement9:      **if**
$\sum_{i=1}^{n_{j}}b_{ij}\leq B_{j}$
**then** //the remaining budget of $\tau_{j}$ is sufficient10:       $W_{j}\leftarrow W_{j}\cup \left\{w_{i}\right\}$ //allocate task $\tau_{j}$ to crowd workers *i*11: **update**
$\theta_{b},\theta_{t},\theta_{d},\theta_{tr},\theta_{rp}$ by Equation (4) // update the threshold of related attributes12:      **else** // the remaining budget of $\tau_{j}$ is not sufficient13: **update**
$\theta_{b},\theta_{t},\theta_{d},\theta_{tr},\theta_{rp}$ by Equation (4)14: **end if**      **else** //its attributes don’t satisfy threshold requirement15:       **update**
$\theta_{b},\theta_{t},\theta_{d},\theta_{tr},\theta_{rp}$ by Equation (4)16:      **end if**17:    **end if**18:    **end if**19:   **end if**20:    **if**
$w_{i}$ is not in the target area **then**21:      **if**
$pt_{i}<\theta_{p}{}_{t}$ //malicious competition22:       **continue**23:      **end if**24:      **if**
$pt_{i}\geq \theta_{p}{}_{t}$
**then**
25:        return to the 4–18 steps26:      **end if**27:     **end if**28:  **end for**29: **end for**

In Algorithm 2, a fixed good reputation threshold is set in this paper. According to whether the user’s reputation is greater than the threshold, the determination of payment is divided into two situations. When a crowd worker arrives, if the crowd worker’s reputation value is greater than the threshold (lines 2), the payment of the corresponding task can be obtained before performing this task (lines 3–5). And after finishing this task, the reputation value and trust degree (lines 6–8) will be updated according to the quality of the sensing data. If the user’s reputation value is less than the threshold (lines 12), the payment for the crowd worker will be determined according to the quality certification result after uploading the sensing data to the platform (lines 13–17), and then the crowd worker’s reputation and trust degree will be also updated accordingly (lines 18). Therefore, the high-reputation crowd worker can obtain the required payment, but the low-reputation crowd workers will get the payment less than the bidding price, and their reputation and trust degree will be reduced. This can motivate users to work hard to complete tasks and improve sensing data quality to get more payments.

 **Algorithm 2** Payment Determination **Input:**
$w_{i}$’s bidding profile $F_{i}$, each task’s quality value $q_{ij}$, the system threshold of good reputation $\theta_{rp}^{good}$ **Output:** Payment $p_{ij}$1: **for**
i←1
**to**
m
**do**2: **if**
$rp_{i}\geq \theta_{rp}^{good}$
**then** //$w_{i}$’s reputation satisfies the system threshold of good reputation3:  **for**
j←1
**to**
$n_{i}$
**do**4:     $p_{ij}=b_{ij}$ //$w_{i}$ will get payment equal to the value of the bidding price before performing $\tau_{j}$5:     pay for $w_{i}$6:     **if**
$\tau_{j}$ be finished **then**7:     Quality certification8:     Update $rp_{i}$ and $tr_{i}$ by Equations (7)–(11)9: **end if**10: **end for**11: **end if**12: **if**
$rp_{i}<\theta_{rp}^{good}$
**then** //$w_{i}$’s reputation doesn’t satisfy the system threshold of good reputation13: **for**
j←1
**to**
$n_{i}$
**do**14: **if**
$\tau_{j}$ be finished **then**15:     Quality certification16:     $p_{ij}=b_{ij}\cdot (\lambda \cdot e^{q_{ij}}-1)$ //$w_{i}$ will get payment which determined based on the quality of sensing data after submitting sensing data and quality certification17:     pay for $w_{i}$18:     Update $rp_{i}$ and $tr_{i}$ by Equations (7)–(11)19:     **end if**20: **end for**21: **end if**22: **end for**

### 3.3. Mechanism Design against Free-Riding

In our incentive mechanism, we consider three free-riding behaviors of crowd workers. The first is the malicious competition on bidding price. The second is the malicious bidding to disrupt the system order. The third is that they submit bidding profile, but do not earnestly perform the task. This paper gives corresponding incentive and punishment strategies for these three free-ridding behaviors:

(1) For the malicious competition on bidding prices, we give a cost estimation method for crowd workers to calculate the reasonableness of the bidding price submitted by crowd workers. In order to recruit enough crowd workers to participate the sensing tasks, this paper publishes tasks to target area and surrounding areas to encourage mobile users to perform tasks in target areas. Therefore, the cost for crowd workers to perform a sensing task contains the cost of moving and task-sensing. The farther the crowd worker is from the target area, the higher the moving cost. The moving cost of $w_{i}$ is calculated by Equation (14):
  (14) $$c_{m}=\kappa d_{i}\;$$
  where κ is crowd worker’s unit movement cost. The task-sensing cost of $w_{i}$ is calculated by Equation (15).
  (15) $$c_{s}=\mu t_{i}{}_{j}\;$$
  where μ is crowd worker’s unit sensing cost, and $t_{ij}$ is the sensing time that $w_{i}$ performs $\tau_{j}$. The total cost for $w_{i}$ to perform $\tau_{j}$ is defined by Equation (16):
  (16) $${\mathrm{cost}}_{ij}=c_{m}+c_{s}\;$$
  In order to give crowd workers a reference for bidding and increase the likelihood of successful bidding, we will give a reference bidding price based on moving distance and sensing time of the crowd worker which is calculated by Equation (16). Then the crowd worker can determine their bidding price based on the reference value.
(2) For identifying the crowd workers with other two free-ridding behaviors during the bidding process, and effectively select the high-quality crowd workers to improve the efficiency of MCSs, we divide mobile users into following types based on their locations and possible behaviors
  (A) Mobile users are in surrounding of the target area.
    (a) Mobile users who may go to the target area before task deadline:
      (i) the mobile users are interested in the task of target area, and submit their bidding profiles.
      (ii) the mobile users are not interested in the task of target area, thus, they will not participate in the auction.
      (iii) the mobile users are not interested in the task of target area, but they only want to try to participate in the auction or deliberately disturb the system order. Furthermore, they will submit false sensing data if they are selected.
    (b) Mobile users who are unlikely to go to the target area before task deadline.
      (iv) the mobile users are similar to (ii).
      (v) the mobile users are similar to (iii).
  (B) Mobile users in the target area.
    (vi) the mobile users are interested in the task, and submit their bidding profile to the platform.
    (vii) the mobile users are similar to (ii).
    (viii) the mobile users are similar to (iii).

From the above analysis, we can see that our goal is to select the right crowd workers among the mobile users mentioned in (i) and (vi). However, it still cannot thoroughly exclude the free-riders in MCSs. Therefore, we pay for crowd workers based on their reputations, that is to say, the crowd workers with high reputation will get payment before performing tasks, otherwise, they will get payment based on their data quality after submitting sensing data. Then, based on the quality certification result of the sensing data submitted by crowd workers as well as bidding price and task value, we update their reputation and trust degree to encourage them to submit high-quality sensed data in lower bidding price. For the mobile users described in (iii) and (viii), we comprehensively consider their attributes when receiving the bidding profiles, if they are judged as the free-riders, they will be rejected by the platform, and their attribute values cannot affect the threshold updating. In addition, if some malicious users are selected as the crowd workers in auction improperly, we will decrease their trust degree and reputation so that they will be immediately rejected in the next auction. We will reject the mobile users described in (v) based on their attributes because they are judged that they will not go to the target area and perform the task. Therefore, their attributes will not affect the threshold updating. The mobile users described in (ii), (iv) and (vii) cannot affect the system, but it is necessary to adopt an appropriate incentive strategy to inspire them to participate in the crowd tasks. 

### 3.4. Analysis of the Proposed Incentive Mechanism

In this section, the five properties of the proposed incentive mechanism are proved.

**Lemma** **1.** *The proposed incentive mechanism satisfies computational efficiency.*

**Proof.**  If the number of bidders is *m*, and the number of sensing tasks submitted by the mobile user is *n*, the time complexity of the for-loop in the algorithm of the crowd worker selection is at most *O*(*mn*). The complexity in payment determination algorithm is still *O*(*mn*). Therefore, the time complexity of our proposed incentive mechanism is *O*(*mn*), that is, the whole process of our proposed incentive mechanism can be completed in polynomial time.

**Lemma** **2.** *The proposed incentive mechanism satisfies individual rationality.*

**Proof.**  There are three possible bidding results for $w_{i}$. First, if $w_{i}$ fails to bid for $\tau_{j}$, then $c_{ij}=0$, $p_{ij}=0$, we obtain $u_{ij}=p_{ij}-c_{ij}=0$. Second, $w_{i}$ successfully bid for $\tau_{j}$ and whose reputation satisfies $rp_{i}\geq \theta_{rp}^{good}$, then $w_{i}$ will get payment $p_{ij}=b_{ij}$ before performing $\tau_{j}$. Because $b_{ij}>c_{ij}$, we obtain $u_{ij}=p_{ij}-c_{ij}>0$. The third case is $rp_{i}<\theta_{rp}^{good}$, $w_{i}$ will receive payment $p_{ij}=b_{ij}\cdot (\lambda \cdot e^{q_{ij}}-1)$ after completing $\tau_{j}$. According to $\frac{c_{ij}+b_{ij}}{b_{ij}}\cdot \frac{1}{e^{q_{ij}}}\leq \lambda \leq \frac{2}{e^{q_{ij}}}$, we can get $u_{ij}=p_{ij}-c_{ij}\geq 0$. Therefore, our proposed incentive mechanism satisfies individual rationality.

**Lemma** **3.** *The proposed incentive mechanism satisfies budget-balance.*

**Proof.**  Because $P_{j}\leq B_{j}$ for $\tau_{j}$, and $B_{j}<v_{j}$, we can get $v_{j}-P_{j}>0$ and $U=\sum_{\tau_{j}\in \Gamma_{i}(W_{j})}v_{j}-\sum_{\tau_{j}\in \Gamma_{i}(W_{j})}P_{j}$. Therefore, the platform will gain non-negative utility, and our proposed incentive mechanism satisfies budget-balance.

**Lemma** **4.** *The proposed incentive mechanism is truthful.*

**Proof.**  The proposed incentive mechanism considers user’s multiple attributes when selecting crowd workers, and judges the rationality of user’s bidding price through estimating their cost. The user’s related attribute values will be updated based on their performance after submitting sensing data. In order to increase the success rate in auction, users will choose to bid truthful. Therefore, our proposed incentive mechanism is truthful.

**Lemma** **5.** *The proposed incentive mechanism is honest.*

**Proof.**  Our multi-attribute reverse auction takes into account multiple attributes of users in the auction process, and estimates the costs of users based on the sensing time of users and the distance to the target area. Then the platform judges the reasonableness of user’s bidding in order to avoid malicious competition on bidding price. In addition, we also consider multiple attributes such as user’s location, trust degree, reputation, and the possibility that a crowd worker moves to the target area to ensure that the crowd worker can honestly complete the task.

## 4. System Performance Evaluations

In this section, the performance of the proposed incentive mechanism is evaluated through simulation experiments. 

### 4.1. Simulation Setup

Our experiments all run on the Windows 7 operating system with an Intel(R) Core(™) i5-6500 CPU @ 3.20 GHz, 8.00 GB memory and the PyCharm 2017.1.4 simulation platform. Each experimental result is the mean of 100 runs. We adopted a real-world dataset which called Foursquare to emulate the locations of crowd workers and calculate the real distance based on the location [44]. Furthermore, we calculate the moving cost and sensing cost based on the distance and sensing time. Then we set a bidding price range for crowd workers based on the distance and sensing time, and the bidding price of $w_{i}$ for $\tau_{j}$ is randomly taken from the range. Because our algorithm considers multiple attributes and the real-world datasets can’t satisfy it, we simulate the other parameters, which are shown in Table 2. Specifically, the parameters $t_{ij}$, $rp_{i}$, and $td_{i}$ are taken from the normal distribution random numbers within the interval shown in Table 2. $T_{j}$ is the total sensing time of $\tau_{j}$ which is randomly taken from the interval shown in the Table 2. $B_{j}$ is the budget of $\tau_{j}$, which is a random number that is randomly taken from the table’s range. The parameter $n_{i}$ is the number of tasks that $w_{i}$ is interested in. 

We compare our proposed multi-attribute reverse auction algorithm with the general auction algorithm and two-stage auction algorithm to evaluate the performance of our proposed algorithm. The general auction algorithm has a fixed price threshold and the worker will be accepted only if his bidding price is not higher than this threshold, otherwise he will be rejected. The two-stage auction algorithm rejects the first batch of workers which are used as the sample, and decides the price threshold based on these samples. We utilize the same experimental parameters and the experimental environment for comparison experiments to guarantee the truthfulness and fairness of the experiments.

### 4.2. Simulation Results

In order to verify the effectiveness of the multi-attribute reverse auction algorithm, we prove the efficiency of different algorithms for selecting crowd workers by calculating the increase speed in total payments paid to crowd workers. The *x*-coordinate represents the number of bidders increasing with time, and the *y*-coordinate denotes the value of P(W). Under different budgets, the faster increase speed the algorithm is, the higher the efficiency of the algorithm is. Correspondingly, the changes of the system average trust degree after accepting a crowd worker under different parameters are evaluated. The *x*-coordinate also represents the number of bidders increasing with time. The *y*-coordinate represents the average trust degree of the system. We verify the effect of different parameters on the efficiency of the algorithm and the trust degree of the system through controlling variable method. The variables parameters for each change are P(W), m, ρ, $n_{i}$ respectively.

**Figure 4:**
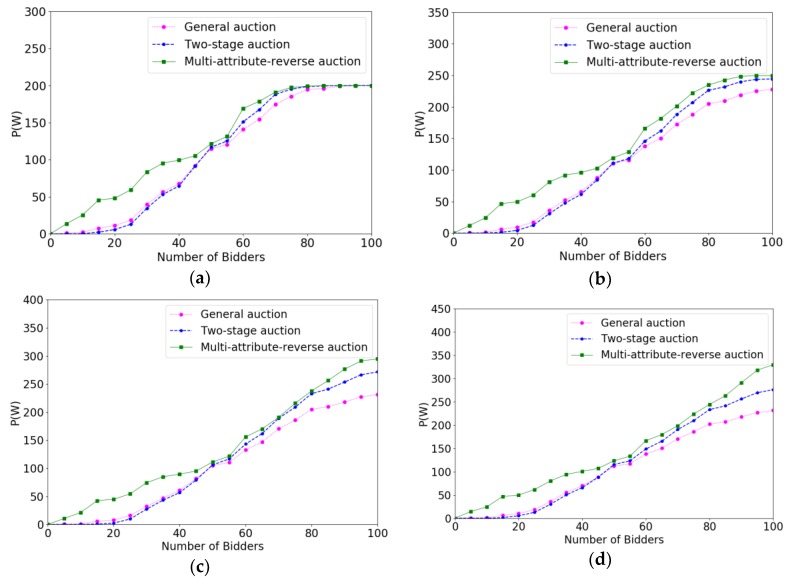
The comparison results of efficiencies when P(W) varied from 200 to 350 with the increment of 50. (**a**) P(W)=200; (**b**) P(W)=250; (**c**) P(W)=300; (**d**) P(W)=350.

Figure 4 show the experimental result of efficiencies when the budget varied from 200 to 350 with the increment of 50. From Figure 4a,d, we can see that the system reached the target value gets later with the budget grows, which is because only more bidders can consume more budget. The process of online auction algorithm is that system determines immediately whether accepts a bidder as the bidder submits the bidding profile, so the value of budget will not influence the bidders who are arriving at the system early. However, for the bidders who are arriving later, they will have more possibility to be accepted if the budget is sufficient. It is clear that multi-attribute reverse auction has maintained a rapid growth rate compared to two-stage auction and general auction, which because the threshold of multi-attribute reverse auction algorithm changes dynamically and has a good adaptability. Specifically, we can see that before the 45 rounds, the general auction algorithm is better than the two-stage auction algorithm because the two-stage auction algorithm rejects some early coming users as a sample, so that they need more time to select crowd workers. However, if the number of bidders and budget is sufficient, this effect will become smaller and smaller and the two-stage auction is better than a general auction after 45 rounds. 

**Figure 5:**
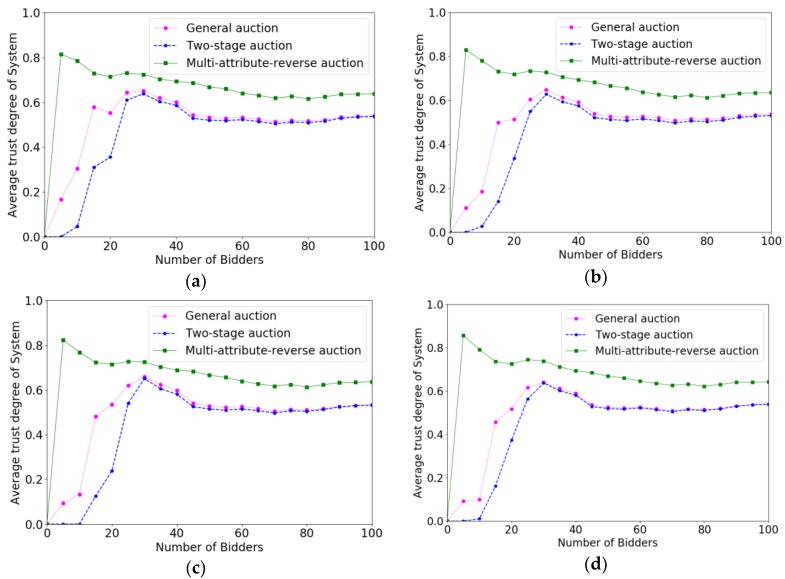
The comparison results of trust degree of system when P(W) varied from 200 to 350 with the increment of 50. (**a**) P(W)=200; (**b**) P(W)=250; (**c**) P(W)=300; (**d**) P(W)=350.

Figure 5 shows the comparison results of trust degrees for different auction algorithms with the changes of P(W). From Figure 5a–d, we can see that the overall trust degree of system has not changed much with the change of P(W), which is because the degree of trust of the system is only influenced by the trust degrees of crowd workers and has little relation with P(W). It is evident that the average trust degree of the crowd workers selected in the multi-attribute reverse auction algorithm is higher than that of general auction algorithm and two-stage auction algorithm, while the average trust degree of system is almost the same with two-stage auction algorithm and general auction algorithm. It is because that multi-attribute reverse auction algorithm considers multiple attributes including trust degree when selecting crowd workers, while general auction algorithm and two-stage auction algorithm only consider the single attribute of bidding price.

**Figure 6:**
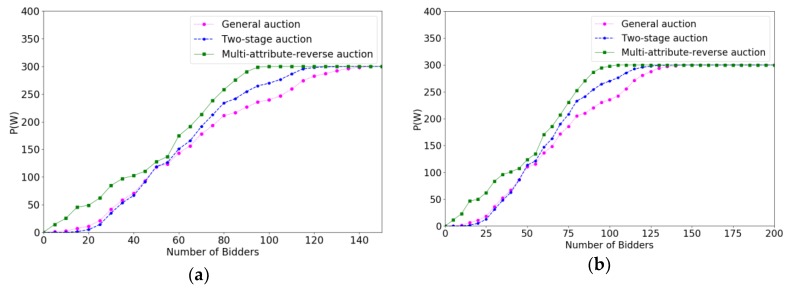

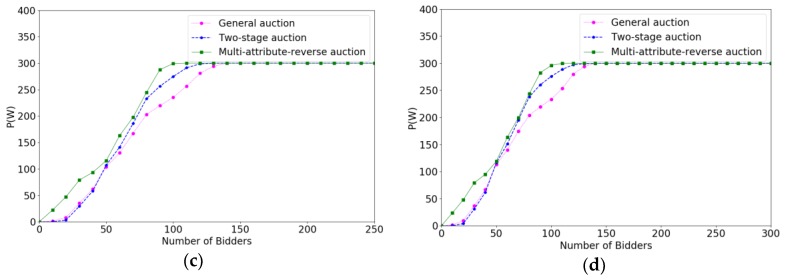
The comparison results of efficiencies when m varied from 150 to 300 with the increment of 50. (**a**) m=150; (**b**) m=200; (**c**) m=250; (**d**) m=300.

Figure 6 show the experimental result of efficiencies when the number of bidders is varied from 150 to 300 with the increment of 50. From Figure 6a–d, we can see that the curves of the three algorithms are almost the same, which occurs because the system will not accept any bidder when the budget reaches 300 and the three algorithms reached the target value at about 140 rounds. Due to the character of the online auction algorithm, the selecting speed of the three auction algorithms are only influenced by the bidding price and other attributes of bidders and not influenced by the value of m. We can see from any of the four figures that multi-attribute reverse auction algorithm always produced a faster selection speed than other two algorithms, because the thresholds of multi-attribute reverse auction algorithm are dynamic, and the value of initial threshold has a smaller impact on multi-attribute reverse auction algorithm than general auction algorithm and two-stage auction algorithm.

**Figure 7:**
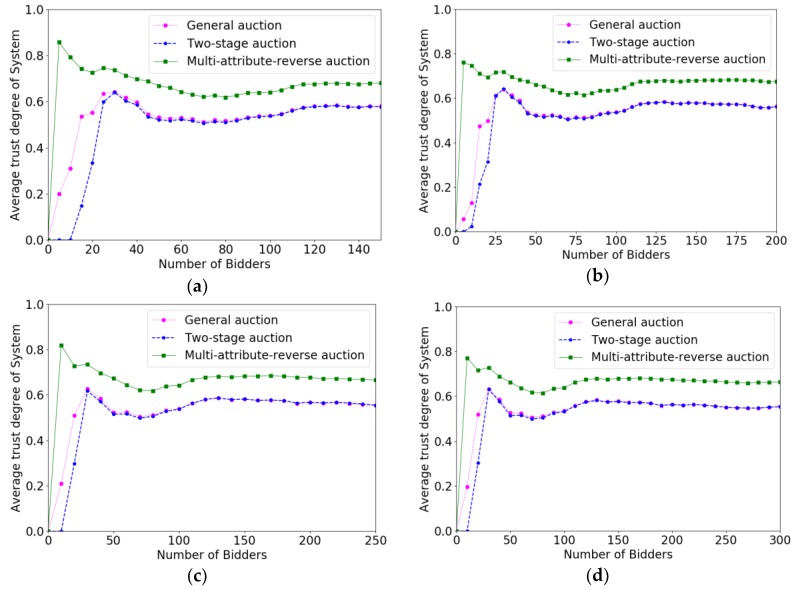
The comparison results of trust degree of system when m varied from 150 to 300 with the increment of 50. (**a**) m=150; (**b**) m=200; (**c**) m=250; (**d**) m=300.

As can be seen in Figure 7, the comparison results of trust degrees of the system for different auction algorithms with the changes of m has slight changes because the trust degree of the system is only influenced by the trust degree of bidders. Around the 70th round, the average trust degree of the system reduces to the minimum, which means that the crowd worker’s attribute value in this part is slightly lower, and the multi-attribute reverse auction algorithm requires some time to adjust attributes’ thresholds. The three algorithms are roughly the same in the overall change trend of trust degrees, but the overall value of the multi-attribute reverse auction algorithm is about 0.15 higher than the trust degrees of two-stage auction algorithm and the general auction algorithm. The average trust degrees of the three algorithms tend to be a stable value after some changes with the increase of m.

**Figure 8:**
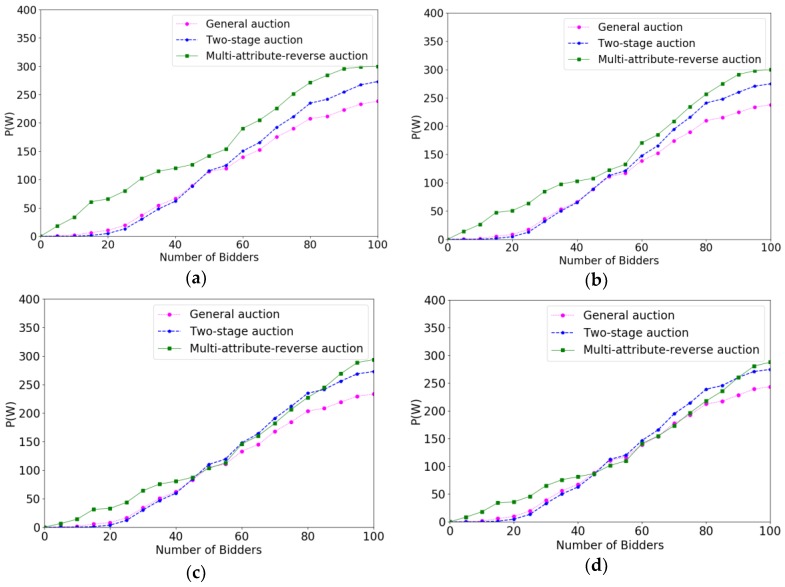
The comparison results of efficiencies when ρ varied from 0 to 0.09 with the increment of 0.03. (**a**) ρ=0; (**b**) ρ=0.03; (**c**) ρ=0.06; (**d**) ρ=0.09.

Figure 8a–d shows the effect of ρ on the efficiencies under multi-attribute reverse auction algorithm. The efficiencies of the algorithm become worse and worse as ρ increased when ρ increased more than 0.06. We know that ρ is a system parameter that adjusts the threshold change. What we hope is that the threshold will be adjusted according to overall condition of crowd workers’ attributes and the differences between different bidders is not too large in general. Therefore, when ρ is too large, the attribute value of the individual crowd worker will make the threshold change a lot and the change of the threshold cannot adapt to the overall situation of the crowd workers.

**Figure 9:**
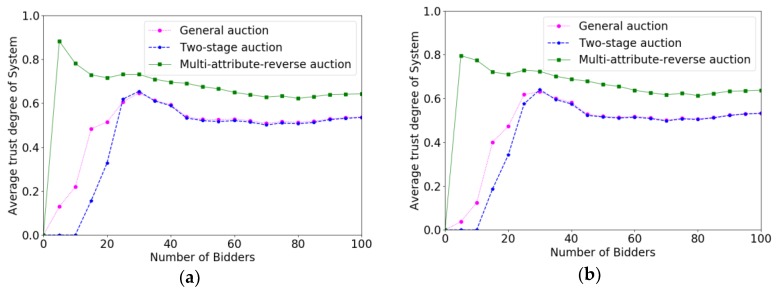

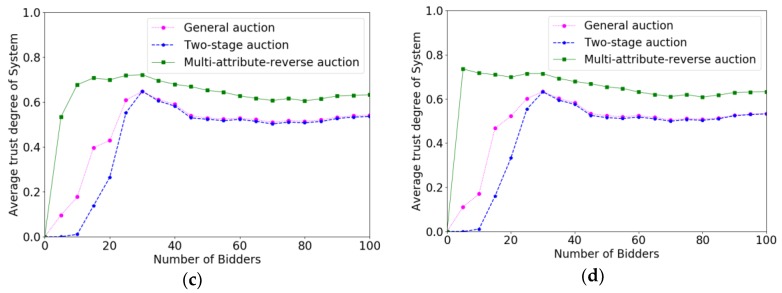
The comparison results of the trust degree of system when ρ varied from 0 to 0.15 with the increment of 0.05. (**a**) ρ=0; (**b**) ρ=0.03; (**c**) ρ=0.06; (**d**) ρ=0.09.

Figure 9a–d shows the effect of ρ on the trust degree of system under multi-attribute reverse auction algorithm. The trust degree of system under this algorithm becomes worse and worse as ρ increased. However, it mainly reflects when the system selecting crowd workers at the very beginning and as the number of crowd workers increases, the impact of ρ on average trust degree of system becomes smaller.

**Figure 10:**
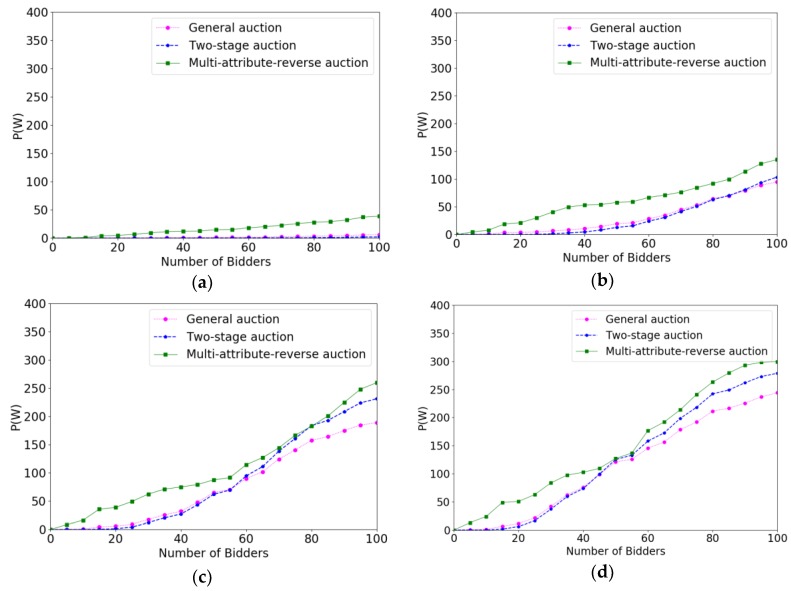
The comparison results of efficiencies when the interval of $n_{i}$ varied from 1 to 13 with the increment of 4, (**a**) $n_{i}=1$; (**b**) $n_{i}\in [1,5]$; (**c**) $n_{i}\in [1,9]$; (**d**) $n_{i}\in [1,13]$.

Figure 10 shows the comparison results of efficiencies when the interval of $n_{i}$ changes from 1 to 13. As the crowd workers’ maximum number of bidding tasks increase from 1 to 13, the efficiencies of the three algorithms better and better. The reason is that the algorithm will get more bidders’ information to calculate their threshold and the chances of bidding success are getting bigger as crowd workers bidding for more tasks, so the efficiencies of the algorithms become better accordingly. Although the efficiencies of the three algorithm all become better with the increasing of $n_{i}$, the multi-attribute reverse auction always performs better than two-stage auction and general auction due to its good adaptiveness.

**Figure 11:**
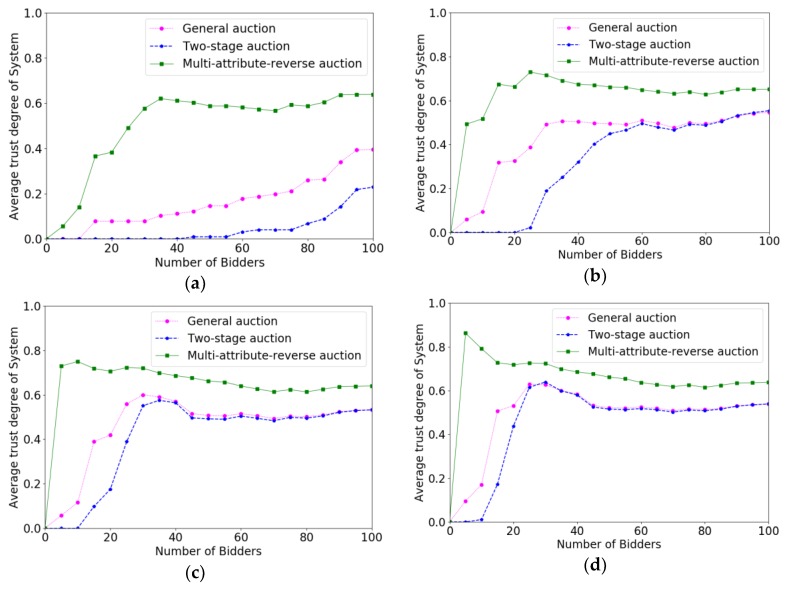
The comparison results of trust degree of system when the interval of $n_{i}$ varied from 1 to 13 with the increment of 4. (**a**) $n_{i}=1$; (**b**) $n_{i}\in [1,5]$; (**c**) $n_{i}\in [1,9]$; (**d**) $n_{i}\in [1,13]$.

Figure 11 shows the comparison results of trust degree when the interval of $n_{i}$ changes from 1 to 13. We can see from Figure 11a–d, that the changes of $n_{i}$ have a significant influence on the two-stage auction algorithm and general auction algorithm. This is because both the two-stage auction and the general auction are fixed threshold algorithms and they have less adaptability than a multi-attribute reverse auction. As for the two-stage auction, when crowd workers bid for less tasks, the sample of the task will get less, and the threshold of two-stage auction may not appropriate. Therefore, the both two algorithms have not selected eligible users at beginning of the auction.

The above experimental analysis shows that no matter how the parameters change, multi-attribute reverse auction algorithm always have better performance. The trust degree of the crowd workers is always the highest, and the threshold can be changed according to the overall situation of the users which prove that our proposed algorithm has better adaptability.

## 5. Conclusions

In this paper, we design an online incentive mechanism based on multi-attribute reverse auction for MCSs. The mechanism considers the requirement of task requester and avoid malicious competition through considering multiple attributes of crowd workers when selecting them. What’s more, we divide payment determination into two situations to inspire crowd workers to improve their reputation by providing high quality sensing data. We have proved that our proposed online incentive mechanism satisfies the following properties: computational efficiency, individual rationality, budget-balance, truthfulness and honesty. Simulation results show that our proposed can improve the efficiency and trust degree of system. In future works, we will consider both the social relationships between crowd workers in mobile crowd sensing networks. In addition, the spatio-temporal attribute will be further researched when establishing incentive mechanism.

## Figures and Tables

**Table 1:** Frequently used notations.

Notation	Description
$n_{j},n_{i}$	The crowd worker number of $\tau_{j}$, the task number of $w_{i}$
$rp_{i},tr_{i}$	The reputation of $w_{i}$, the trust degree of $w_{i}$
$W_{j}$	The crowd worker set of $\tau_{j}$
$B_{j}$	The budget of $\tau_{j}$
$n_{i}^{good},n_{i}^{bad},n_{i}^{total}$	The good quality task number of $w_{i}$, the bad quality task number of $w_{i}$, the total task number of $w_{i}$
$q_{ij}$	The sensed data quality of $w_{i}$ for $\tau_{j}$

**Table 2:** Simulation settings.

$t_{ij}\;$	$rp_{i}\;$	$td_{i}\;$	$T_{j}\;$	$B_{j}\;$	*n*	ρ	$n_{i}\;$	*m*	P(W)
[1, 17]	[0, 1]	[0, 1]	[20, 25]	[25, 35]	50	0.05	[1, 12]	100	300
